# Editorial: Exploring the association between metabolism and psychiatric disorders

**DOI:** 10.3389/fpsyt.2024.1456763

**Published:** 2024-07-11

**Authors:** Anna-Rita Atti

**Affiliations:** Department of Biomedical and Neuromotor Sciences, University of Bologna, Bologna, Italy

**Keywords:** metabolism, folate deficiency, hyperhomocysteinemia, ketogenic diet, mitochondrial dysfunction, antipsychotics, multidisciplinary research, personalized medicine

## Introduction

The complex and bidirectional relationship between metabolism and mental health is well known: metabolic abnormalities can influence the onset and progression of psychiatric disorders, and psychiatric disorders can contribute to metabolic dysregulation. Addressing the interplay between metabolism and mental health requires an integrated approach focused on genetics, lifestyle, and medication management. So far, there is limited literature exploring the association between metabolism and psychiatric disorders in detail. The aim of this editorial is to shed light on this issue by summarizing the publications included in the current Research Topic titled “Exploring the Association between Metabolism and Psychiatric Disorders.” Overall, the four studies included reinforce the need for multidisciplinary research at both the preclinical and clinical levels, as well as collaborative care. Only a holistic approach will guarantee the possibility of improving patients’ quality of life.

## Current research

The goal of this Research Topic is to use a broad approach to explore the association between metabolism and a variety of psychiatric disorders, unravel new insights into the underlying mechanisms of these conditions, and help identify new targets for treatment.

Frajerman et al. investigated the prevalence of folate deficiency, vitamin B12 deficiency, and hyperhomocysteinemia in hospitalized patients with psychotic disorders aged 15–30 years. Deficiencies in folate, vitamin B12, and hyperhomocysteinemia were common: 38% in participants with first-episode psychosis, 27% in those affected by schizophrenia, and 36% in subjects with schizoaffective disorders. The most frequent abnormality was folate deficiency, with lower levels found in men compared to women. Furthermore, the deficiency was correlated with more severe disorders. Antipsychotic dosage was positively associated with B12 levels and negatively with homocysteinemia. The authors suggest folate supplementation as a possible personalized complementary approach to treating psychotic disorders, even at early stages.

A few preclinical studies have demonstrated the favorable role of induced ketosis—either through a Ketogenic Diet (KD) or exogenous supplementation with ketone bodies (KBs)—in alleviating neuropsychiatric symptoms. The review by Omori et al. describes the putative neuroprotective effects of KBs on inflammasomes, as well as their role in promoting neurogenesis in the central nervous system. Additionally, the use of KD to prevent antipsychotic-induced hyperglycemia is described and proposed as a complementary treatment option. Despite the promising results and the relative simplicity of either KD or KB supplementation, the therapeutic effectiveness remains to be established due to the lack of well-designed and well-powered human studies. Further clinical research is warranted.

Second-generation antipsychotics are well known to induce severe weight gain, type 2 diabetes, and cardiovascular disease. The mini-review by Mortimer et al. hypothesizes that mitochondrial dysfunction might trigger metabolic dysregulation. The proposed mechanisms include the dysregulation of glucose and fatty acid metabolism, an increase in reactive oxygen species and oxidized proteins, and effects on electron transport chain complexes. Despite scant—and sometimes conflicting—evidence, this body of knowledge is of paramount importance for the goal of personalized medicine.

## Future directions and conclusions

In conclusion, the Research Topic “Exploring the Association between Metabolism and Psychiatric Disorders” includes articles that highlight the importance of multifaceted research and an evidence-based clinical approach. Concentrating on research into dysmetabolism is pivotal for enhancing the health outcomes of patients affected by psychiatric disorders. Dietary prescriptions are increasingly used because of their favorable risk/benefit ratio. However, they should be administered under the guidance of a trained medical professional, and only effective remediations should be prescribed. Therefore, preclinical and clinical research aimed at identifying effective intervention strategies and treatments is mandatory.

Future studies should focus on elucidating the precise mechanisms through which metabolic dysregulation affects psychiatric conditions and identifying specific biomarkers that can predict the development of metabolic abnormalities in patients with psychiatric disorders. This will provide a deeper understanding of the bidirectional relationship between metabolism and mental health, potentially leading to the development of targeted interventions that can improve both metabolic and psychiatric outcomes. To enhance the generalizability of research findings, future studies should include larger and more heterogeneous sample sizes, encompassing diverse populations across different age groups, ethnicities, and socio-economic backgrounds.

To conclude, the papers included in this Research Topic support an integrated model of psychiatric disease, encompassing both biological (e.g., genetic, developmental, and inflammatory) and psychosocial issues. This holistic approach enhances our understanding of the complex interplay between metabolism and mental health, ultimately guiding the development of more effective, personalized treatment strategies that address the multifaceted needs of patients with psychiatric disorders.

## Author contributions

A-RA: Writing – original draft, Writing – review & editing.

